# FC-98 Regulates TLR9-Mediated of CXCL-10 Expression in Dendritic Cells via MAPK and STAT1 Signaling Pathway

**DOI:** 10.1155/2014/926130

**Published:** 2014-02-17

**Authors:** Yonghong Yang, Huan Dou, Xiaoqin Li, Yuxian Song, Wei Gong, Renxiang Tan, Yayi Hou

**Affiliations:** ^1^The State Key Laboratory of Pharmaceutical Biotechnology, Division of Immunology, Medical School, Nanjing University, Nanjing 210093, China; ^2^Jiangsu Key Laboratory of Molecular Medicine, Nanjing 210093, China; ^3^Institute of Functional Biomolecules, School of Life Sciences, Nanjing University, Nanjing 210093, China

## Abstract

Dendritic cells (DCs), as the most potent professional antigen presenting cells, play a crucial role in both innate and adaptive immune systems. Genomic bacterial DNA mimicked by unmethylated CpG motifs is discovered to possess immunostimulatory effects. CpG-DNA recognized by Toll-like receptor 9 (TLR9) on DCs arouses many immune diseases (such as cancer, viral infection, and autoimmune disorders). In this study we investigated the effects of FC-98 on CpG-induced bone marrow-derived DCs (BMDCs). The results showed that FC-98 significantly inhibited the CpG-induced BMDCs maturation and function by suppressing the expression of surface markers (CD40, CD80, CD86, and MHCII). Moreover, FC-98 downregulated the expression of C-X-C motif chemokine 10 (CXCL-10) both at the mRNA and protein level after CpG induction. Meanwhile, FC-98 markedly affected the migration of BMDCs to T cells without affecting their endocytosis capacity. Furthermore, FC-98 was confirmed to decrease CXCL-10 expression by inhibiting CpG-induced activation of MAPKs (ERK, JNK, and p38) and STAT1 signaling. Overall, these results suggested that FC-98 was a potential molecule in the treatment of CXCL-10-mediated immune diseases.

## 1. Introduction

Dendritic cells (DCs) are the most potent professional antigen presenting cells and play a crucial role in both innate and adaptive immune systems [1]. Indeed, there is compelling evidence that DCs participate in the induction and treatment of numerous diseases, such as autoimmune diseases [2, 3], cancer [4], and infection [5]. DCs are derived from bone marrow progenitor cells and exist in vivo at two different stages: immature and nature. In the steady state, most DCs are immature [6]. When exposed to stimuli, DCs capture antigens and migrate to draining lymph nodes, where they present antigens to T lymphocytes. This process involves endocytosis, the upregulation of cell-surface molecules markers (CD40, CD80, CD86, and MHCII) [7], and the large quantity synthesis of chemokines and cytokines [8]. Moreover, DCs can recognize and response to pathogen-associated molecular patterns through pattern recognition receptors including Toll-like receptors (TLRs) family [9]. To date, 10 TLRs in human and 13 TLRs in mouse have been identified, respectively [10]. Binding of ligands to TLRs results in maturation of DCs and initiation of the adaptive immune responses [11]. Recently genomic bacterial DNA mimicked by unmethylated CpG motifs is discovered to possess immunostimulatory effects [12]. CpG-DNA receptor, Toll-like receptor 9 (TLR9), is also expressed by DCs. TLR9 locates in endosomes and is responsible for sensing unmethylated CpG-DNA, discriminates between self- and foreign DNA, and plays an important role in many immune diseases such as autoimmune diseases and cancer [13]. Synthetic oligodeoxynucleotides (CpG-ODN) have been found taking part in promoting activation and maturation of bone marrow-derived dendritic cells (BMDCs) [14].

It was reported that the expression of C-X-C motif chemokine 10 (CXCL-10) by DCs is elevated following CpG-ODN stimulation [15, 16]. CXCL-10, also named as interferon gamma-induced protein 10 (IP-10), is a member of CXC chemokines and known as activator on antitumor, antiviral, antifungal, and autoimmune diseases [17–19]. CXCL-10 also plays an important role in the interaction between DCs and T cells through binding and activating its receptor CXCR3, expressing on the surface of T cells [20]. Thus the interaction influences the recruitment and activation of Th1-polarized cells [21]. Recently, more and more attention was paid to the potential of CXCL-10 signaling pathway. In human macrophages, microglia, and epithelial and cancer cells, CXCL-10 expression has been demonstrated to be mediated through MAPK (ERK1/2, JNK, and p38) and STAT1 signaling pathway [22–24]. However, the regulation pathway of CXCL-10 in BMDCs is still not clear.

Our previous study demonstrated that FC-98, a benzenediamine derivate, inhibited the TNF-*α*, IL-6, MCP-1, and NO production from LPS-induced murine macrophages through attenuating the activation of NF-*κ*B, JNK, and IRF3 signaling pathway. Moreover, FC-98 effectively improved the survival rate and injured organ of mice's sepsis model (unpublished). However, it is still unclear whether FC-98 has effects on DCs and the underling mechanisms. In this study, we attempted to investigate the effects of FC-98 on the maturation of CpG-induced BMDCs and their functions. We showed that FC-98 markedly inhibited the expression of costimulatory molecules of BMDCs without affecting their endocytosis capacity. Moreover, FC-98 significantly inhibited the expression of CXCL-10 both on mRNA and protein level, leading to decreasing migration ability of T cells to DCs. As expected, FC-98 blocked both MAPK and STAT1 signaling pathway upstream the CXCL-10. For the first time FC-98 was approved to be a potent compound for treatment of TLR9-mediated and CXCL-10-assosiated autoimmune and inflammatory diseases.

## 2. Materials and Methods

### 2.1. Reagents, Antibodies, and Mice

**Figure 1:**
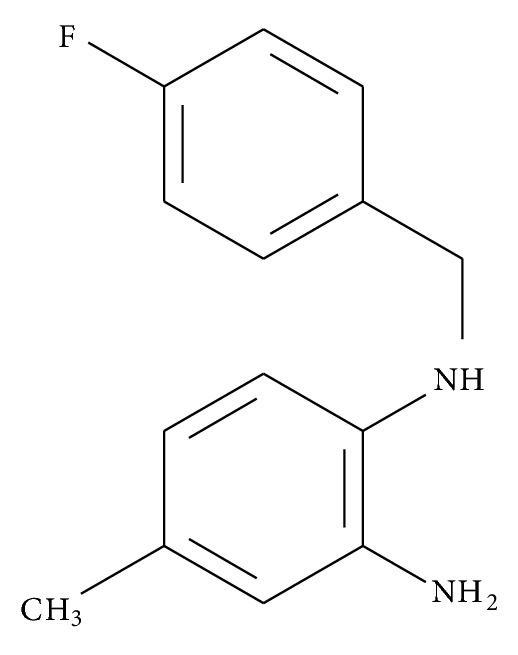
Stereostructure of structure of FC-98.

FC-98 (N^1^-[(4-fluorophenyl)methyl]-4-methyl-1,2-benzenediamine, purity >99%, chemical structure shown in Figure 1) was commercially synthesized as previously reported [25]. For testing, FC-98 was dissolved in dimethyl sulfoxide (DMSO) at a concentration of 50 mM as a stock solution and diluted to the desired concentration with medium before each experiment. RPMI 1640, fetal bovine serum (FBS), penicillin, and streptomycin were purchased from Gibco Inc. (Grand Island, NY, USA). Recombinant granulocyte-macrophage colony-stimulating factor (GM-CSF) was from Miltenyi Biotec (Bergisch Gladbach, Germany), and recombinant interleukin-4 (IL-4) was from PeproTech, Inc. (Rocky Hill, NJ). CpG-ODN 1668 was synthesized by Invitrogen (Shanghai, China). FITC Annexin V Apoptosis Detection Kit was purchased from BD Pharmingen (Heidelberg, Germany). Cell counting kit-8 (CCK-8) was purchased from DojinDo Laboratories (Kyushu, Japan). Mouse CXCL-10 enzyme-linked immunosorbent assay (ELISA) kit was purchased from R&D Systems (Minneapolis, MN, USA). Antibodies (Abs) used to detect the expression of CD11c, CD40, CD80, CD86, and MHCII as well as isotype-matched control mAbs were purchased from eBioscience (San Diego, CA, USA). FITC-dextran was from Sigma-Aldrich (St. Louis, MO, USA). BCA kit was purchased from Pierce (Rockford, IL). Anti-mouse JNK, phospho-JNK, p38, phospho-p38, ERK, phospho-ERK, STAT1, phospho-STAT1, Tublin, and GAPDH for Western blot analysis were purchased from Cell Signaling Technology (Danvers, MA, USA). Female C57BL/6 mice 4–6 weeks old were purchased from the Animal Research Center of Yangzhou University (Yangzhou, China). All of the animals were maintained under specific pathogen-free conditions and acclimatized for at least 1 week to the surrounding environment prior to use. Experiments were conducted according to institutional animal ethics guidelines.

### 2.2. Generation of Mouse Bone Marrow Derived Dendritic Cells

Bone marrow cells were obtained from the tibiae and femurs of C57BL/6 mice and treated with red blood cell lysing buffer. Cells were cultured in RPMI-1640 medium supplemented with 10% FBS, 100 *μ*g/mL penicillin, 100 *μ*g/mL streptomycin, 10 ng/mL murine GM-CSF, and 1 ng/mL IL-4 at a density of 1 × 10^6^ cells/mL. The plates were incubated at 37°C in 5% CO_2_ for 7 days and half of the medium was replaced by fresh medium containing GM-CSF and IL-4 on days 3 and 5. At day 7, nonadherent and loosely adherent cells were harvested and transferred into new culture plates for further experiments.

### 2.3. Cell Viability Assay

The cytotoxicity of FC-98 was evaluated using the Cell Counting Kit 8 (CCK-8) according to the manufacturer's instructions. In brief, 100 *μ*L of 5 × 10^3^ DCs was seeded in 96-well plates and incubated overnight and cells were then incubated with various concentrations of FC-98 for 24 h. Subsequently, 10 *μ*L of the CCK-8 solution was added to each well and incubated for another 3 h. The absorbance at 450 nm was measured using microplate reader (Synergy HT, Bio-Tek).

### 2.4. Assessment of Apoptosis of DCs

The extent of apoptosis in DCs was assayed by the Annexin V/PI staining. In brief, DCs were cultured with various concentrations of FC-98 for 24 h at 37°C. After washing twice with cold medium phosphate buffered saline (PBS), cells were incubated with Annexin V-FITC in dark for 5 min, and then PI was added before analysis of samples with flow cytometry. Flow cytometry was performed by using FACS Calibur system (BD Biosciences, Franklin Lakes, NJ, USA). And the data were analyzed by WinMDI (Scripps Institute, La Jolla, CA).

### 2.5. Flow Cytometric Analysis of Surface Markers

After treatment, DCs were washed twice in FACS medium containing 1% FCS and 0.1% NaN_3_. Subsequently, cells were incubated for 30 minutes at 4°C with appropriately diluted antibodies of CD80, CD86, CD40, and MHCII according to the standard procedure. Cells were washed and fixed in 4% paraformaldehyde. An isotype control was used for each antibody. Fluorescence was measured using flow cytometry detection, and data were analyzed using Cell Quest software (BD Biosciences, Franklin Lakes, NJ, USA). The results of all experiments were expressed as mean fluorescent intensity (MFI).

### 2.6. Endocytosis Assay

Assay of DCs endocytotic activity was measured by uptake of FITC-dextran and quantitated using flow cytometry. In brief, 2 × 10^5^ DCs were incubated with various concentrations of FC-98 for 2 h, and then FITC-dextran (final concentration 0.5 mg/mL) was added into the culture medium at 37°C in dark for 1 h. Then cells were collected and washed 3 times with cold PBS to stop endocytosis. Trypan blue (1.2 mg/mL) was used to quench the endocytic activity and remove any free FITC-dextran. Cells were analyzed by flow cytometric analysis. A parallel experiment performed at 4°C served as a negative control.

### 2.7. RNA Isolation, Reverse Transcription Polymerase Chain Reaction, and Q-PCR

Total RNA was extracted from 2 × 10^6^ cells using TRIZOL (Invitrogen, USA) following the manufacturer's description. The RNA quantity was determined by spectrophotometer at 260 nm. 20 *μ*L cDNA was synthesized from 1 *μ*g total RNA using a revert aid first-strand cDNA synthesis kit (Fermentas). Quantitative real-time PCR analysis was performed in 20 *μ*L PCR reaction mixture using the 7300 PCR System (Life Technologies, Grand Island, NY, USA). Amplification conditions were 95°C for 10 min, and 40 cycles of 95°C for 30 s, 60°C for 30 s, and 72°C for 30 s. The mRNA levels for target gene were normalized to *β*-actin in the same sample. The primer sequences used in this study were as follows: CXCL-10, 5′-CCAAGTGCTGCCGTCATTTTC-3′ (forward) and 5′-GGCTCGCAGGGATGATTTCAA-3′ (reverse); GAPDH, 5′-GGTGAAGGTCGGTGTGAACG-3′ (forward) and 5′-CTCGCTCCTGGAAGATGGTG-3′ (reverse).

### 2.8. ELISA Assay

BMDCs were treated as experiment designing. Supernatants were harvested after 24 h culture and assayed for CXCL-10 expression according to manufacturer's instructions. In brief, cell culture supernatants and CXCL-10 standards in triplicate were added to precoated 96-well plate and incubated for 2 h at 22–25°C. Afterward, the plate was washed 5 times with washing buffer. Then, HRP-conjugated secondary antibody was added and the plate was incubated for 2 h at 22–25°C. After washing 5 times, substrate solution were added and incubated for 30 min at 22–25°C in the dark. The stop solution was then added to each well and the absorbance was read at 450 nm using a microplate reader.

### 2.9. Chemotaxis Assay

To assess the effect of FC-98 on T cells transmigration to BMDCs, Chemotaxis assay were performed using a 24 well Transwell system with 5 *μ*m pore polycarbonate filter (Corning, NY, USA). T cells were purified from lymph node, and 1 × 10^6^ T cells in 100 *μ*L of RPMI 1640 were added to the upper chamber and 600 *μ*L supernatant of DCs was added to the lower chamber. After 3 h incubation at 37°C, cells that had migrated into lower wells were harvested and counted by flow cytometry.

### 2.10. Western Blot Analysis

About 1 × 10^6^ DCs were lysed in ice-cold buffer (0 mM Tris-HCl (pH 7.6), 250 mM NaCl, 0.5% NP-40, 3 mM EDTA, and 1.5 mM EGTA) containing 10 *μ*g/mL aprotinin, 10 *μ*g/mL leupeptin, 1 mM DTT, 1 mM PNPP, and 0.1 mM Na_3_VO_4_ for 30 min. After centrifugation, cell lysates were collected and measured by the BCA Protein Assay Reagent Kit (Pierce, Biotechnology). Equal amounts of protein from each sample were mixed with 1X loading buffer and denatured at 95°C for 5 min. Then they were subjected to 10% SDS-PAGE and transferred on to polyvinylidene difluoride (PVDF) membranes (Roche, Germany). After the PVDF membranes were blocked for 2 h with 5% bovine serum albumin in 0.1% Tween-20/TBS, the membranes were incubated overnight with primary antibodies at a concentration of 1 : 1000 at 4°C. After washing, the membranes were incubated with HRP-conjugated secondary antibody at 1 : 3000 dilutions for 2 h at the room temperature and then washed again. Protein bands were detected by enhanced chemiluminescence Western blotting detection reagents using ECL method, and images were acquired by FluorChem FC2 System (Alpha Innotech Corporation, USA). The membrane was stripped and reprobed with GAPDH or Tublin antibody to equal loading of proteins.

### 2.11. Assay of Luciferase Reporter Gene Expression

BMDCs were seeded in 24-well plates at 1 × 10^5^ cells per well and incubated overnight. Cells were cotransfected with 100 ng pAP-1 luciferase reporter plasmids and 10 ng pRL-TK-Renilla luciferase plasmids using Lipofectamine LTX reagent. Total amounts of plasmid DNA were equalized with pGL6 (empty control vector). After 24 h of culture, cells were pretreated with FC-98 for 1 h and then stimulated with 1 *μ*M CpG for another 6 h. Luciferase activities were measured using the Dual-Luciferase Reporter Assay system (Promega, Madison, WI) according to the manufacturer's instructions. Data are normalized for transfection efficiency by dividing Firefly luciferase activity with that of Renilla luciferase.

### 2.12. Statistical Analysis

All results were analyzed with Prism 5.0 (GraphPad Software, Inc., San Diego, CA) and expressed as means ± SD Data analysis was done using Student's *t*-test and one-way analysis of variance (ANOVA). Differences with *P* < 0.05 were considered significant.

## 3. Results

### 3.1. FC-98 Had No Influence on BMDCs Viability and Apoptosis

**Figure 2:**
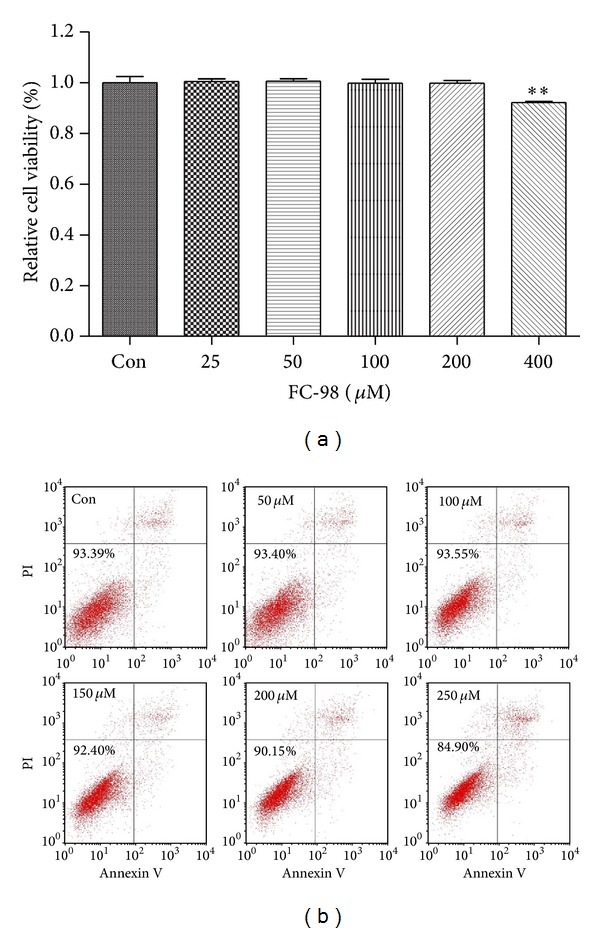
The effect of FC-98 on the cell viability and apoptosis of BMDCs. (a) BMDCs were treated with FC-98 in the range of 0–400 *μ*M for 24 h and then the CCK-8 assay was applied to detect the cell viability. (b) Cells were stained with Annexin V and PI followed by flow cytometry analyses. Cells stained with FITC-Annexin V alone (Annexin V^+^/PI^−^) were considered in early apoptosis, whereas those stained with both FITC-Annexin V and PI (Annexin V^+^/PI^+^) were considered in the advanced stages of apoptosis or necrosis. The FITC-Annexin V and PI double-negative (Annexin V^−^/PI^−^) cells were considered alive. Data are expressed as mean ± SD of three independent experiments. **P < 0.01 versus control (Con) group.

FC-98 has a molecular weight 290 and its stereostructure is shown in Figure 1. In the initial series of experiments, we evaluated the effect of FC-98 on the cell viability of BMDCs by CCK-8 assay. BMDCs were isolated and purified as described in Section 2 and then exposed to various concentrations (0, 25, 100, 200, and 400 *μ*M) of FC-98 for 24 h. The viability of the cells was determined at the wavelength of 450 nm. The results showed that FC-98 did not affect the cell viability up to 200 *μ*M (Figure 2(a)).

To further explore the cytotoxicity of FC-98 on DCs, we performed the Annexin V/PI costaining flow cytometry analysis. BMDCs were harvested and cultured in 24-well plate, and then a range of 0–250 *μ*M FC-98 were added. After 24 h coculture, the cells were washed and stained for Annexin V and PI. Flow cytometry analysis was used to determine the cell viability after FC-98 treatment on DCs. No significant difference in Annexin V^−^/PI^−^ was detected between BMDCs treated with FC-98 at the concentrations from 0 to 200 *μ*M (Figure 2(b)). Taken together, the concentration of FC-98 ≤200 *μ*M was selected for the following experiments.

### 3.2. FC-98 Suppressed the Phenotypic Maturation of CpG-Induced DCs without Influence on Endocytosis

**Figure 3:**
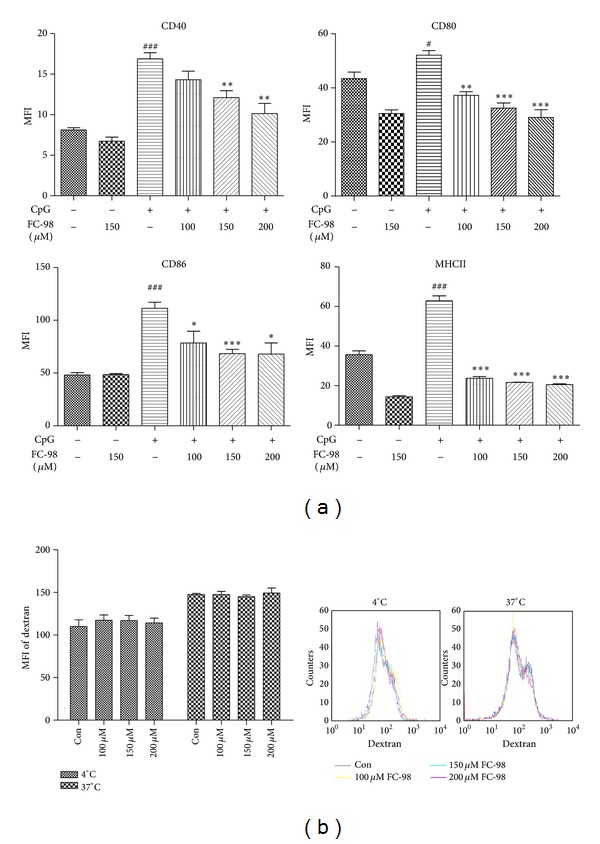
FC-98 inhibited CpG-induced upregulation of surface markers without influence on endocytosis. (a) BMDCs were treated with indicated concentrations of FC-98 for 2 h, and then CpG (1 *μ*M) were added in. After 24 h, the expressions of CD40, CD80, CD86, and MHCII were analyzed by flow cytometry. (b) BMDCs were treated with or without FC-98 and then 0.5 mg/mL FITC-Dextran was added for another 1 h at 37°C (4°C as control) and analyzed by flow cytometry. Data are shown as mean ± SD of three independent experiments. ^#^ *P* < 0.05, ^###^ *P* < 0.001 versus control group; *P < 0.05, **P < 0.01, and ***P < 0.001 versus CpG only group.

When DCs detect exogenous danger such as bacterial DNA, the maturation will be initiated immediately. The matured DCs express high levels of costimulatory molecules (CD40, CD80, and CD86) and MHCII. To determine the effects of FC-98 on the maturation of DCs, immature DCs were exposed to different concentrations of FC-98 for 2 h, followed by 24 h stimulation of CpG (1 *μ*M). The expression of MHCII, CD40, CD80, and CD86 were assayed by flow cytometry. As shown in Figure 3(a), FC-98 alone had little effect on the MFI of MHCII and costimulatory molecules on BMDCs compared with control group. In contrast, these molecules expressions on BMDCs pretreatment with FC-98 were sufficiently reduced, since the expression of surface markers was significantly upregulated with treatment of CpG.

DCs are the most potent professional antigen capturing and presenting cells. Endocytosis is thought to be characteristic during the process of pathogen uptake. To examine the endocytosis of BMDCs, we treated BMDCs as indicated and then used 0.5 mg/mL FITC-labeled Dextran to quantify the endocytic capacity by flow cytometry.  Figure 3(b), the similar endocytic capacity was observed in BMDCs both in 37°C and 4°C after FC-98 treatment. This indicated that FC-98 had no influence on the endocytic function of BMDCs.

### 3.3. FC-98 Suppressed the Expression and Function of CXCL-10 in CpG-Induced BMDCs

**Figure 4:**
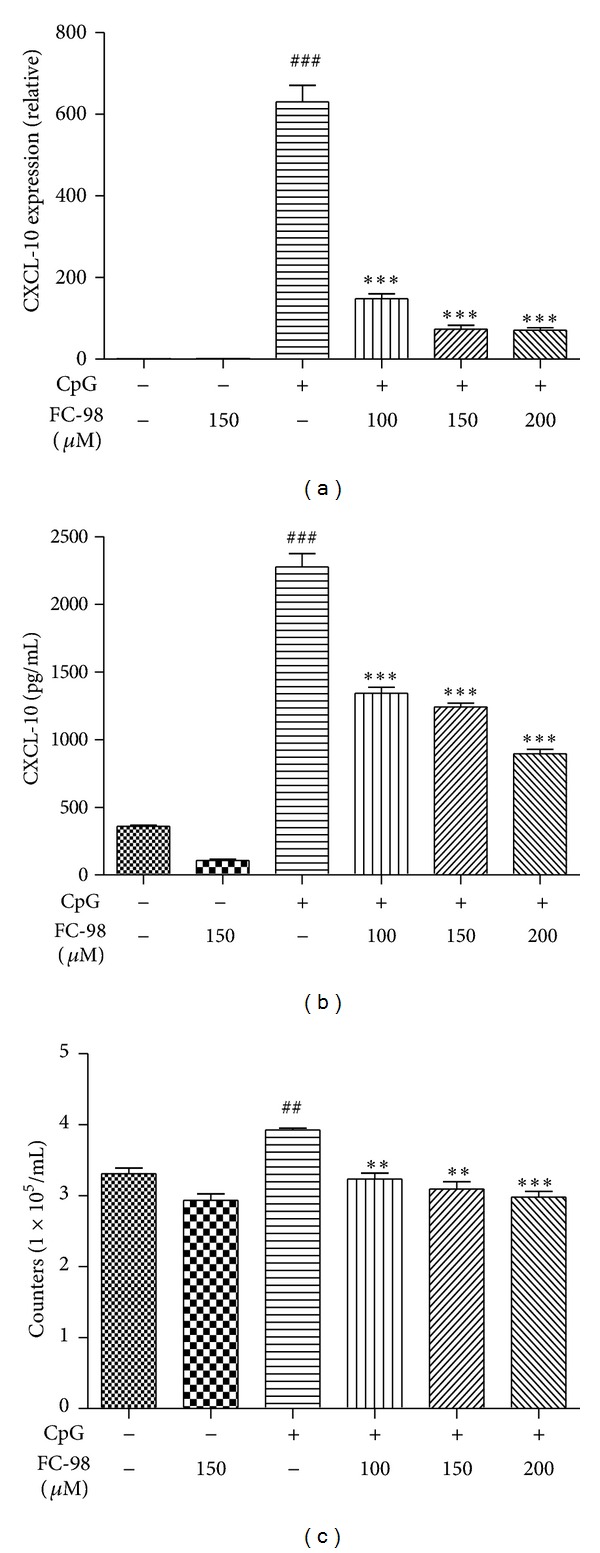
Effect of FC-98 on BMDCs CXCL-10 expression and function. BMDCs were treated with various concentrations of FC-98 for 2 h, and then 1 *μ*M CpG was added for another 6 h before q-PCR (a) or 24 h before ELISA (b). (c) Transwell experiment was performed to examine the function of pretreated BMDCs on T cells. The data are mean ± SD of three independent experiments. ^##^ *P* < 0.01; ^###^ *P* < 0.001 a versus control group; **P < 0.01; ***P < 0.001 versus CpG only group.

CXCL-10 plays an important role in the interaction between DCs with T cells. To further determine the production of CXCL-10 in BMDCs, we treated the cells with various concentrations of FC-98 for 2 h. After stimulation with CpG for additional 6 h, we extracted the total RNA and examined the expression of CXCL-10 on transcription level. As shown in Figure 4(a), FC-98 could markedly decrease the mRNA expression of CXCL-10 in DCs at different dosages. Moreover, we treated cells with CpG for 24 h and the culture supernatants were collected and analyzed by ELISA. The similar results were found in the protein level of CXCL-10 (Figure 4(b)).

Since CXCL-10 plays an important role in the migration of T cell [21], we tried to clarify the effect of FC-98 on the migration of T cells to BMDCs and chemotaxis assay was used. As shown in Figure 4(c), FC-98 alone had little effect on the migration of T cells compared to control group, while all three concentrations of FC-98 markedly inhibited CpG-induced migration of T cells to BMDCs.

### 3.4. FC-98 Inhibited CXCL-10 through MAPK and STAT1 Signaling Pathway

**Figure 5:**
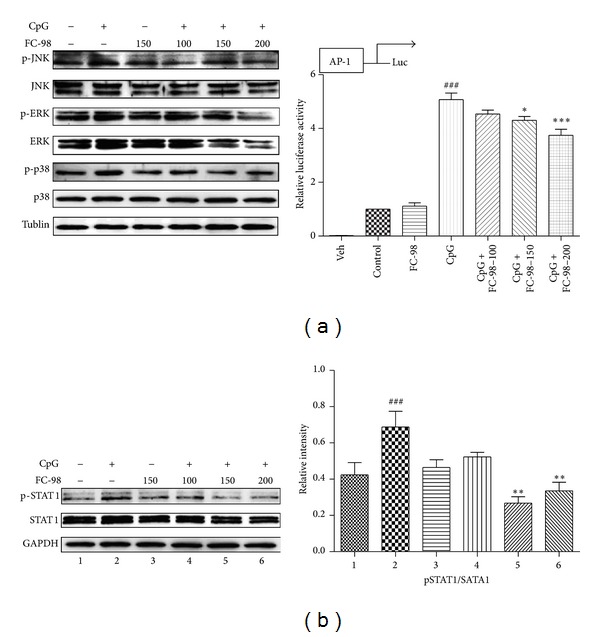
FC-98 inhibited activation of MAPK and STAT1 to downregulate the CXCL-10 expression. (a) Left: BMDCs were pretreated with FC-98 for 2 h, followed by 30 min CpG treatment; the phosphorylation of of MAPK (ERK, JNK, and p38) signaling pathway was analyzed by western blot. The results shown are representative experiments from three independent assays. Right: BMDCs were cotransfected with 100 ng pAP-1 luciferase reporter plasmid and 10 ng pRL-TK-Renilla luciferase. Total amounts of plasmid DNA were equalized using empty control vector. After 24 h of culture, cells were pretreated with FC-98 for 1 h and then stimulated with 1 *μ*M CpG for another 6 h. Luciferase activity was measured and normalized by Renilla luciferase activity. Data are shown as mean ± SD of three independent assays. ^###^ *P* < 0.001 versus control group; *P < 0.05, ***P < 0.001 versus CpG-only group. (b) Left: experiments were duplicated as described in (a), left, and phosphorylated STAT1 (p-STAT1) and total STAT1 were detected by western blot. The results shown are representative experiments from three independent assays. Right: the results of western blot were analysed by Image J, and the relative intensity was showed. ^###^ *P* < 0.001 versus control group; ∗∗*P* < 0.01 versus 2 (CpG-only group).

Several studies reported that CXCL-10 expression is mediated through MAPK (ERK1/2, JNK, and p38) and STAT1 signaling pathway in several cell types [22–24]. However, the regulation of FC-98 in BMDCs is unclear. So the mechanism underlying the inhibitory effect of FC-98 on CXCL-10 was examined by detecting the activation of p38, JNK, ERK, and STAT1. Our previous articles had shown that the phosphorylation of p38, JNK, ERK, and STAT1 was sharply raised at 30 min when stimulated with CpG [16]. Thus BMDCs were treated with 100 *μ*M, 150 *μ*M, or 200 *μ*M FC-98 for 2 h, followed by CpG stimulation at 1 *μ*M for 30 min. Western blot data showed that FC-98 slightly inhibited the phosphorylation of JNK, p38, and ERK1/2 (Figure 5(a), left). To further confirm the inhibitory effects of FC-98 on MAPK signaling activation, BMDCs were transfected with an AP-1-dependent luciferase gene reporter plasmid. As shown in Figure 5(a), right, pretreating cells with FC-98 displayed the reduced CpG-induced luciferase activity, indicating a lower AP-1 activity. As to STAT1 signaling, FC-98 potently inhibited the phosphorylation of STAT1 (Figure 5(b)), whereas FC-98 had little influence on their total protein levels. These findings indicated that FC-98 inhibited the expression of CXCL-10 by reducing the activation of the MAPK and STAT1 signaling cascades in CpG-activated DCs.

## 4. Discussion

In the present study, we for the first time found that FC-98 regulated TLR9-mediated of CXCL-10 expression in BMDCss via MAPK and STAT1 signaling pathway. Using CCK-8 assay and Annexin V/PI double staining, FC-98 below 200 *μ*M had no cytotoxicity on BMDCs. Upon CpG stimulation, FC-98 significantly downregulated the expression of CD40, CD80, CD86, and MHCII on the surface of BMDCs. In contrast, endocytosis of FITC-Dextran in the cells treated with FC-98 had no obvious difference between 4°C and 37°C. Furthermore, FC-98 at different concentrations could markedly decrease the expression of CXCL-10 at both the mRNA and protein level in CpG-induced DCs. Meanwhile, FC-98 also suppressed the migration captivity related to CXCL-10 of T cells to BMDCs. Finally, we found that FC-98 inhibited the activation of MAPK and STAT1 signaling pathway. All these data suggest that FC-98 may be a potential drug in the treatment of CXCL-10 mediated diseases.

DCs are the most potent professional antigen presenting cells and play a crucial role in promoting the natural immune and starting the adaptive immune [26]. DCs are derived from bone marrow progenitor cells and differentiate into immature DCs in lymphoid or nonlymphoid tissues [27]. When appropriate stimulations are identified, immature DCs can upregulate expression of cell surface markers (MHCII, CD40, CD80, and CD86) and proinflammatory cytokines and chemokines [28]. At the same time, their ability of uptaking Ag turns into presenting Ag [29]. Then mature DCs activate T cells and regulate their responses [30]. DCs recognize pathogens by a set of pattern-recognition receptors. Among them, TLRs play an essential role [31]. CpG-ODN, which is similar to the bacterial DNA, can act as the TLR9 ligand to promote maturation and activation of DCs [32]. TLR9 are associated with various diseases such as autoimmune diseases and infectious diseases [33]. To date, TLRs-based therapeutics were more and more used in treatment of diseases [34].

Therefore, it is significant to regulate DCs maturation and function via the agents. Our present study had revealed several small molecule compounds to possess immunomodulatory properties on BMDCs via TLR signaling pathway [16, 35]. Our laboratory had recently demonstrated the small molecule compound FC-98 which can inhibit the expression of TNF-*α*, IL-6, MCP-1, and NO from LPS-induced mouse macrophages through attenuating the activation of NF-*κ*B, JNK, and IRF3 signaling pathway. Moreover, FC-98 can effectively improve the survival rate and injured organ of mice's sepsis mode (unpublished). However, the immunoregulatory roles of FC-98 in the immune system remain needing further confirmation. The effect of FC-98 on the maturation and function of DCs remained unknown. In the present study, we revealed that FC-98 could significantly attenuate the phenotypic and functional maturation of CpG-induced BMDCs. FC-98 could strongly inhibit the expression of MHC and costimulatory molecules (CD40, CD80, and CD86). Costimulatory molecules CD80/CD86 expressed on DCs can provide a co-stimulatory signal to T lymphocytes and induce expansion of antigen-specific T cells [36]. On the other hand, capability to uptake FITC-Daxtran of BMDCs was not affected by FC-98, suggesting that FC-98 might influence BMDCs maturation via other ways not the endocytosis. These results suggested that FC-98 might be a potential inhibitor of DC maturation.

After maturation, DCs produce a serious of chemokines. Among them, CXCL-10 is critical to recruit and activate Th1-polarized cells and has the abilities of antitumor, antiviral, and antifungal [37–40]. In DCs, CXCL-10 production in regulatory DCs can downregulate T-cell response and inhibition of recruited Th1 cell proliferation [41]. CXCL-10 is also critical for rendering a protective cellular immunity during SLA-CpG-DC vaccination that confers protection against *Leishmania donovani *infection [42]. In our study, we found that BMDCs produced large amounts of CXCL-10 upon CpG stimulation and pretreatment of FC-98 could notably impair its upregulation at both mRNA and protein levels.

CXCL-10 is correlated with the tissue infiltration of T lymphocytes and mediates in the recruitment of T cells [43]. To further confirm the inhibitory effect of FC-98 on CXCL-10, we also examined the effect of the BMDCs culture supernatant on the T cells migration. The results showed that FC-98 predominantly inhibited the T cell migration induced by CpG. Thus, it is possible that FC-98 might inhibit the chemokine CXCL-10 expression of DCs, further inhibiting the recruitment of T cells.

As to the mechanisms of FC-98 downregulating CXCL-10 production, we explored the MAPK and STAT1 signaling pathways. Williams R found that the upregulation of CXCL10 at both mRNA and protein levels is mediated by the activation of the p38, JNK signaling pathways, and along with the activation of STAT1 [44]. In murine macrophage-like cells, the upregulation of CXCL10 is associated with the activation of JAK1, JAK2/STAT1, and ERK1/2 [22, 24]. In cancer researches, expression of CXCL10 is mediated through the Raf, PI3K, p38/MAPK, JNK/MAPK, and NF-*κ*B signaling cascades [45]. Taken together, we are interested in the MAPK and STAT1 signaling pathway. We pretreated BMDCs with FC-98 for 2 h and then CpG were added for another 30 min and the cell lysis was collected. Western blot experiment revealed that FC-98 inhibited the phosphorylation of JNK, ERK, p38, and STAT1. Luciferase report assay of AP-1 activation was also applied to confirm the results. Collectively, these findings suggest that FC-98 inhibits the expression of CXCL-10 in CpG-induced BMDCs via the MAPK (JNK, ERK, and p38) and STAT1 signaling pathways.

In conclusion, our present study characterized a small molecule FC-98 regulating BMDCs maturation via CXCL-10. Thus, FC-98 may be potential in the treatment of CXCL-10- associated diseases.
